# SNPexp - A web tool for calculating and visualizing correlation between HapMap genotypes and gene expression levels

**DOI:** 10.1186/1471-2105-11-600

**Published:** 2010-12-17

**Authors:** Kristian Holm, Espen Melum, Andre Franke, Tom H Karlsen

**Affiliations:** 1 Norwegian PSC Research Center, Clinic for Specialized Medicine and Surgery, Oslo University Hospital Rikshospitalet, 0027 Oslo, Norway; 2 Research Institute for Internal Medicine, Clinic for Specialized Medicine and Surgery, Oslo University Hospital Rikshospitalet, 0027 Oslo, Norway; 3 Institute for Clinical Molecular Biology, Christian-Albrechts-University Kiel, Schittenhelmstr. 12, D-24105 Kiel, Germany

## Abstract

**Background:**

Expression levels for 47294 transcripts in lymphoblastoid cell lines from all 270 HapMap phase II individuals, and genotypes (both HapMap phase II and III) of 3.96 million single nucleotide polymorphisms (SNPs) in the same individuals are publicly available. We aimed to generate a user-friendly web based tool for visualization of the correlation between SNP genotypes within a specified genomic region and a gene of interest, which is also well-known as an expression quantitative trait locus (eQTL) analysis.

**Results:**

SNPexp is implemented as a server-side script, and publicly available on this website: http://tinyurl.com/snpexp. Correlation between genotype and transcript expression levels are calculated by performing linear regression and the Wald test as implemented in PLINK and visualized using the UCSC Genome Browser. Validation of SNPexp using previously published eQTLs yielded comparable results.

**Conclusions:**

SNPexp provides a convenient and platform-independent way to calculate and visualize the correlation between HapMap genotypes within a specified genetic region anywhere in the genome and gene expression levels. This allows for investigation of both cis and trans effects. The web interface and utilization of publicly available and widely used software resources makes it an attractive supplement to more advanced bioinformatic tools. For the advanced user the program can be used on a local computer on custom datasets.

## Background

According to dbSNP build 131 [1] more than 14 million single nucleotide polymorphisms (SNPs) have been identified and are annotated as validated [2]. This dense map of human genetic variation has paved the way for the design of genotyping arrays with genome-wide coverage approaching nearly 100% as measured according to a linkage disequilibrium ≥ 0.8. versus all HapMap phase II genotypes [3]. Widespread application of these genotyping arrays in case-control genome-wide association studies (GWAS) have revealed more than 2830 robust associations between genetic variants and a variety of diseases and human phenotypes [4,5]. In most cases, the functional implications of the identified variants with regard to gene expression and protein function remain poorly defined. In some of these cases, altered gene expression has been proposed to serve as the causative mechanism [6-9].

Gene expression levels may be considered a quantitative trait that is influenced by genetic variation and amenable to genetic mapping by means of SNP correlation statistics. Studying this correlation is called expression Quantitative Trait Locus (eQTL) mapping, and has proven to be a useful tool to detect regions and variants of importance to gene expression and thus also in raising hypotheses for the underlying mechanisms of genetic findings in GWAS [6,7,9,10]. The efficiency of the eQTL approach has inspired the implementation of a variety of software tools for the generation of eQTL results for different tissues in multiple species [11,12].

While powerful for the computationally skilled user, most tools do not allow for fast and immediate assessment of a region or gene of interest. eQTL viewer [12] is a customizable tool for plotting eQTL results where the user must provide and prepare his own source data, requiring knowledge of Perl, XML and database querying using SQL. Another tool, FastMap [11], is a Java program that must be installed and run on a local computer. It is intended for groups working with inbred mouse strains and the need to calculate genome-wide eQTL maps. eQTL browser [13] summarizes the putative eQTLs identified in several other studies, but does not allow the user to browse every SNP in a region.

Genome-wide SNP genotypes and gene expression levels from lymphoblastoid cell-lines from the HapMap project are publicly available [14-16]. We wanted to combine the information in these data sets and create an easily accessible web tool where users with no knowledge on programming and complex data handling can visualize the correlation between each SNP within a specified genetic region anywhere in the genome and the expression level of a single gene of interest.

## Implementation

SNPexp http://tinyurl.com/snpexp is implemented as a server-side script, written in Perl 5.10 [17] executing on an Apache HTTP server 2.2 [18]. It takes advantage of the quantitative association test in the whole genome association analysis toolset PLINK [19] for calculation of correlation statistics and the web resource UCSC Genome Browser [20] for visualization of the results. In addition, the entire sourcecode are available to the user and can be customized to run locally on other data sets than the HapMap.

### Source data

#### Genotypes

The HapMap phase II release 23 data set consists of 3.96 million SNP genotypes from 270 individuals from 4 populations (CEU: 90 (30 trios) Utah residents with ancestry from northern and western Europe; CHB: 45 unrelated Han Chinese in Beijing; JPT: 45 unrelated Japanese in Tokyo; YRI: 90 (30 trios) Yoruba in Ibadan, Nigeria) [16]. The data was downloaded as PLINK-formatted binary files, coded according to NCBI (build 36) coordinates for the forward strand, from the PLINK web site [21]. In addition, a filtered HapMap phase III release 3 with 1.46 million quality controlled SNPs was also downloaded [22]. The genetic model under which the SNP genotypes operate in influencing gene expression will vary between different SNPs and transcripts. To open up for all possible genetic models, SNPexp can analyse SNPs under an additive, dominant, recessive or genotypic model assumption, however for the general first screen of a gene region we recommend the additive model.

#### Expression

Expression levels for 47294 transcripts from EBV-transformed lymphoblastoid cell lines from the same 270 Hapmap individuals are also available [15]. Each gene was represented on the array (Illumina Human WG-6 Expression BeadChip v1) by one or more different transcript probes. This expression data was downloaded from the Genevar web site [23] as two distinct set of files. In the first set each HapMap population (CEU, CHB, JPT, YRI) had been normalized independently (to preserve any population-specific differences). In the second set all populations had been pooled together before normalization, which makes direct comparisons across populations possible.

## Construction

**Figure 1:**
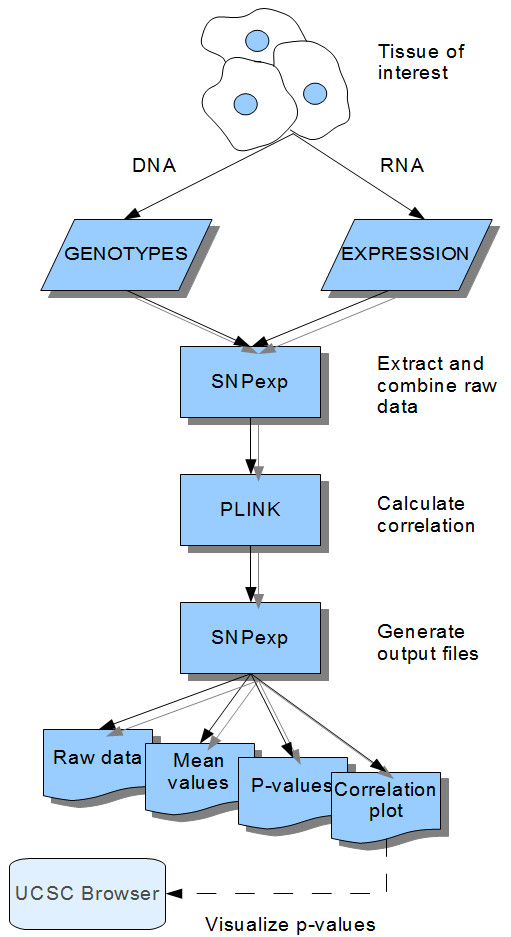
**Workflow stack**. SNPexp combines HapMap SNP genotype data with gene expression data from lymphoblastoid cell lines from the same individuals and automatically applies linear regression and the Wald test in PLINK for assessment of transcript level associations. Data is presented in the form of downloadable text files and the (-log_10_) p-values can be directly visualized in the UCSC Genome Browser.

Figure 1 shows the workflow of the SNPexp tool. It first searches and extracts expression data for the on-chip transcript probe(s) that represented the gene and then uses PLINK to extract genotype data from a specified genomic region. These two data sets are subsequently combined into a new PLINK input file containing both the extracted genotypes and the expression level for each individual in the population. The combined data are instantly analyzed by performing linear regression and the p-values obtained by the Wald test as implemented in PLINK. If a gene was represented by more than one probe on the array, SNPexp runs a separate analysis for each probe, returning one result per probe. The user is advised to judge the statistical results returned with caution as the number of tests performed can be high. To facilitate interpretation in light of multiple testing several methods for correction of the P-values are implemented (Bonferroni, Holm, Sidak, Benjamini&Hochberg and Benjamini&Yekutieli FDR).

Several downloadable files are generated. First, a file formatted for upload as a "custom track" on the UCSC Genome Browser [24] to visualize the p-values (expressed as the negative decadic logarithm) for the correlation between each SNP within the genomic region and the expression level of the gene is created. For multi-probe genes, the result for each probe is displayed as parallel tracks. Both the adjusted and unadjusted p-values are plotted as parallel tracks. A direct link that automatically uploads and plots the result on the UCSC Genome Browser is provided on the SNPexp result page. Secondly, files with the extracted SNP genotypes and the resulting per-SNP genotype frequencies, mean expression levels and both unadjusted and adjusted p-values from the quantitative association test are generated. A comprehensive log file with all output from the various steps in the process is available on the result page.

## Results and discussion

**Figure 2:**
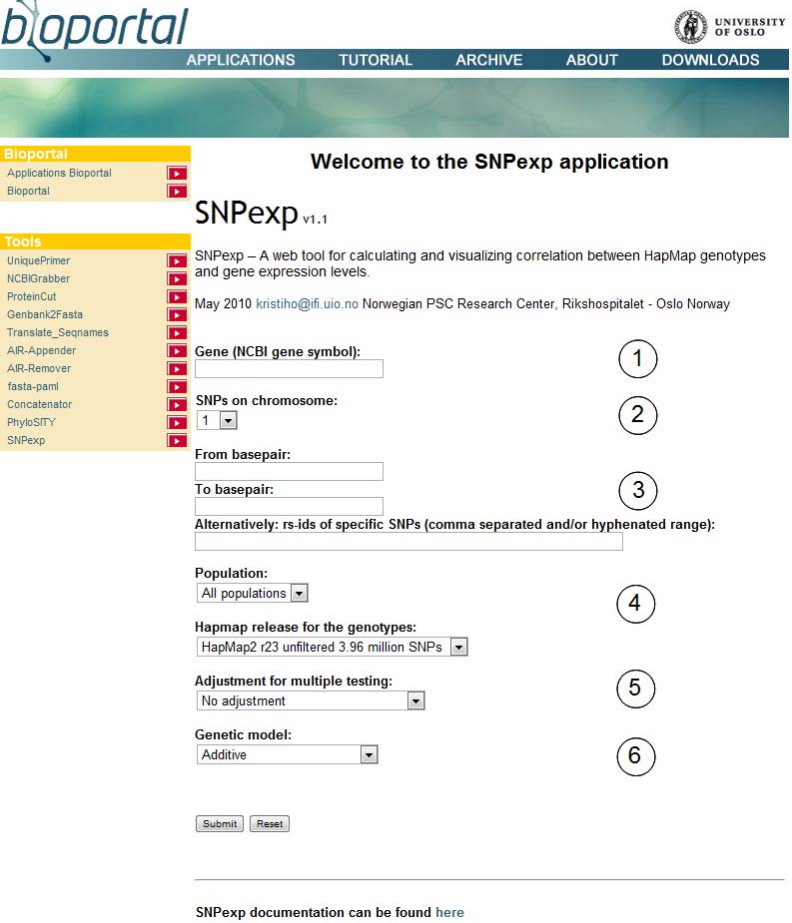
**Screenshot of SNPexp web tool**. User input to SNPexp requires (1) the NCBI gene symbol, (2) a chromosome, (3) a specific genomic region (or specific SNPs) within that chromosome and, (4) the HapMap version and population for which the analysis is to be done, (5) whether to adjust for multiple testing and (6) the assumed genetic model.

Figure 2 shows the front page of the SNPexp web tool. User input to SNPexp requires (1) the NCBI gene symbol, (2) a chromosome, (3) a specific genomic region (or specific SNPs) within that chromosome, (4) the HapMap version and population for which the analysis is to be done, (5) whether to adjust for multiple testing and (6) the assumed genetic model. Pooled assessment of all HapMap populations is also available. Importantly, the utilization of the UCSC Genome Browser for data presentation allows for dynamic interaction, a quick insight into the overall features of the genetic region and multiple customized views. The detailed results files can be taken onward for data presentation using other tools or further analyses.

We advise that the various results files (*extracted_snps_with_expression_valuesPROBE.ped, pvaluesPROBE.linear.assoc.txt, pvaluesPROBE.linear.assoc.adjusted.txt, PROBE.qassoc.means.txt and customtrack.txt *where PROBE refers to the transcript targetID found on the expression array) are downloaded and evaluated along with the log-file. The locally saved customtrack-file can later be uploaded and viewed on the UCSC Genome Browser.

**Figure 3:**
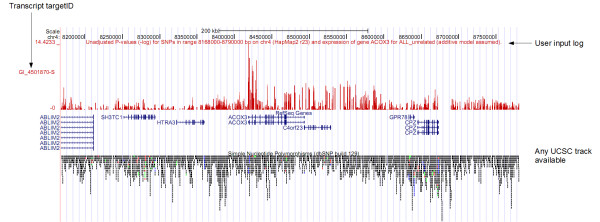
**Visualization of SNPexp results**. The per-SNP calculated (-log_10_) p-values for the correlation (all 210 unrelated HapMap phase II individuals pooled together) between SNPs in a genetic region on chromosome 4 (8,168,000-8,790,000 bp) and the expression of gene ACOX3 as visualized on the UCSC Genome Browser. This plot shows previously published eQTL results from [9] (strongest SNP present in both plots: rs827000 p < 10^-12^). The browser can be customized in different ways to provide gene annotations and SNP positions for the plot.

To assert the validity of SNPexp we specifically aimed to reproduce previously published eQTL results. In particular, the study by Veyrieras et al [9] is based on the same raw HapMap data, meaning that results should therefore be very similar. Figure 3 shows the resulting plot from SNPexp for the correlation between SNPs in a genetic region on chromosome 4 (8,168,000-8,790,000 bp) and the expression of gene *ACOX3 *(for all 210 unrelated HapMap phase II individuals pooled together, analyzed with the additive model without correction for multiple testing). This plot is similar (referenced on the opposite strand) to the plot of the same region in [9] and shows previously published eQTL results (strongest SNP present in both plots: rs827000 p < 10^-12^). Small differences might be caused by filtering of SNPs in [9], normalization methods and differences between Hapmap phase II Release 21 and Release 23.

A genome-wide association study for asthma by Moffat et al [25] is also based on data from lymphoblastoid cell lines, but in another (non HapMap) study population (994 childhood onset asthma patients, 1243 controls; 317000 SNPs genotyped using the illumina Sentrix HumanHap300 BeadChip; Gene expression levels measured with Affymetrix HG-U133 Plus 2.0 chips). The study is of general and particular interest, since eQTL mapping helped to resolve a susceptibility region with strong linkage disequilibrium (LD). We ran SNPexp for the *ORMDL3 *locus against SNPs in the surrounding region on chromosome 17q21. In this assessment, only partial overlap was observed for the most strongly associated SNPs in the eQTL mapping and several exclusive associations was detected using either approach. These apparent discrepancies are not surprising given likely differences between the asthma population and HapMap in genetic constitution as well as with regard to linkage disequilibrium patterns.

SNPexp is created with the intention of being a fast and user-friendly, readily available web tool to analyze and visualize the correlation between two high-quality and publicly available data sets. We decided to use source data as-is, with no additional quality filters applied to neither SNPs nor genes, thereby providing a complete and unbiased set of results which is left to the researcher to further inspect and interpret.

Since the number of possible gene vs. SNP combinations is extremely high and the true model for an allelic effect on gene expression may differ from gene to gene, SNPexp supports the option of using either an additive, dominant, recessive or genotypic genetic model assumption. The pragmatic approach of using the built in Wald test for quantitative traits in PLINK was chosen. While this test is applicable for most purposes, we advice in-depth statistical validation of results from SNPexp that are taken onward to a publishable conclusion or further experiments. The advanced user with knowledge of Perl programming may want to download an "offline version" of the script from the tool's help page, set it up locally and do adaptions to support other data sources etc.

## Conclusions

By combining publicly available HapMap genotype and gene expression data we have developed an interactive web tool (SNPexp) where the user can visualize the correlation between SNP genotypes within a specified genetic region anywhere in the genome and expression levels for a gene of interest. The SNPs and the gene encoding the transcript may reside on separate chromosomes, thereby supporting searches for both cis- and trans-acting eQTLs. The quick and convenient user interface which require minimal computer knowledge and no preparation of source data makes SNPexp an attractive supplement to more advanced eQTL tools.

## Availability and requirements

Project name

SNPexp

Project home page

http://tinyurl.com/snpexp

(Alias for: http://app3.titan.uio.no/biotools/tool.php?app=snpexp).

Operating system

Platform independent

Programming language

Perl 5.10

License

Public Domain

## Authors' contributions

KH implemented the software, performed eQTL mapping and wrote the manuscript. EM participated in the design of the project and helped writing the paper. AF and THK designed and supervised the project and contributed to the manuscript. All authors approved the final manuscript.
